# Sensitivity and Reliability Analysis of Ultrasonic Pulse Parameters in Evaluating the Laboratory Properties of Asphalt Mixtures

**DOI:** 10.3390/ma16216852

**Published:** 2023-10-25

**Authors:** Xiaoshu Tan, Chunli Wu, Liding Li, He Li, Chunyu Liang, Yongchao Zhao, Hanjun Li, Jing Zhao, Fuen Wang

**Affiliations:** 1 College of Transportation, Jilin University, Changchun 130025, China; tanxs20@mails.jlu.edu.cn (X.T.); clwu@jlu.edu.cn (C.W.); lihe326532558@163.com (H.L.); liangcy@jlu.edu.cn (C.L.); 2 Jilin China Railway Expressway Co., Ltd., Changchun 130052, China; 1593266955m@sina.cn (Y.Z.); lihanjun@crecg.com (H.L.); 18813098377@163.com (J.Z.); wangfuen@crecg.com (F.W.)

**Keywords:** asphalt mixture, performance evaluation, ultrasonic testing technology, ultrasonic pulse velocity, grey correlation analysis, dynamic modulus

## Abstract

The ultrasonic test is a promising non-destructive testing technique for evaluating the properties of asphalt mixtures. To investigate the applicability and reliability of ultrasonic testing technology (UTT) in evaluating the performance of asphalt mixtures, ultrasonic tests, indirect tensile tests, compression tests, and dynamic modulus tests were carried out at various temperatures. Subsequently, the distribution characteristics of ultrasonic traveling parameters for asphalt mixtures were analyzed. The variation of ultrasonic pulse velocity and amplitude in dry and wet states with temperature was studied. Then, the correlation between the ultrasonic parameters and both the volume parameters and the mechanical performance parameters of asphalt mixtures was revealed, and the functional relationship between ultrasonic pulse velocity and compressive strength was established. Finally, the reliability of predicting high-frequency dynamic modulus by ultrasonic velocity was verified. The laboratory tests and analysis results indicate that both ultrasonic pulse velocity and amplitude in dry and wet conditions show a decreasing trend with an increase in temperature. Ultrasonic parameters are greatly influenced by asphalt content and mineral aggregate content of 9.5~13.2 mm and 13.2~16 mm. The dynamic modulus at a high-frequency load can be predicted by using ultrasonic velocity, and predicting the results for OGFC and SMA mixtures deduced by using the UPV at a high-frequency load have higher reliability.

## 1. Introduction

Ultrasonic testing technology (UTT) has been widely adopted in the performance evaluation of pavement materials due to its nondestructive characteristics. The ultrasonic test is important not only for quickly obtaining material performance parameters but also for reducing parallel testing errors. A large number of studies on stiffness modulus, dynamic modulus, damage, crack, segregation, fatigue, and healing time of asphalt mixtures adopted the ultrasonic pulse parameters (UPP), which has gradually attracted the attention of pavement engineering researchers [1,2].

Some studies pointed out that there was a strong correlation between the UPP in asphalt mixtures and the indirect stiffness modulus of asphalt mixtures [3,4,5]. In addition, McGovern demonstrated that ultrasonic pulse velocity (UPV) could be adopted to calculate the dynamic modulus of asphalt mixtures under a high-frequency load [6]. Moreover, the dynamic modulus calculated by UPV has a functional relationship with the dynamic modulus obtained by both tension–compression tests [7] and the 2S2P1D (two springs, two parabolic elements, and one dashpot) model [8]. UTT is favorable for determining key rheological parameters (parabolic element parameters and glassy modulus) [9] and improving the prediction accuracy of the dynamic modulus master curve [10].

Additionally, UTT has distinct advantages in assessing the damage and healing performance of asphalt mixtures [11]. Cheng et al. [12] and You et al. [13] used the UTT to evaluate the damage properties of asphalt mixtures subjected to multiple freeze–thaw cycles and multiple coupling factors and concluded that the UTT is a viable method for investigating the potential damage of asphalt mixtures. Pan et al. [14] and Franesqui et al. [15] evaluated the feasibility of the UTT in studying the crack depth of asphalt mixtures and asphalt pavements and reported that the UTT is a promising technology for damage detection. However, it should be noted that the calculated results of crack depth need to be calibrated in the practical application according to the gradation type of asphalt mixtures [15,16]. Not only can using the UTT before and after damage or healing for the same sample save money and time, but it can also provide more comparable and reliable test results.

On the other side, the UTT can also be implemented to investigate the fatigue performance of asphalt mixtures [17]. According to the travel velocity of longitudinal and transverse waves in an asphalt mixture, more performance parameters of asphalt mixtures, such as the Lamé constants, Poisson’s ratio, and modulus, can be deduced. These parameters can then be used to create a function for predicting the fatigue life of asphalt mixtures [18,19], which can benefit the rapid prediction of fatigue life for asphalt mixtures.

As previously stated, numerous studies have demonstrated that UTT serves an incredibly essential function in quickly evaluating asphalt mixture performance. However, the center of all these researches is the reliability and sensitivity of ultrasonic parameters to elaborate the performance of asphalt mixtures. It is well known that the UPV is greatly affected by temperature, material types, material properties, and damage state. Asphalt is a multi-component, temperature-sensitive material with several components. Its internal void, asphalt content, and characteristics all have an influence on ultrasonic travel speed in the asphalt mixture [20]. A key topic that road engineering researchers should explore is how to accurately evaluate the performance of asphalt mixes based on the UPP. However, the influence of a sample’s temperature, dry and saturated states, and gradation on test results is rarely considered in UTTs current research on the evaluation of asphalt mixture performance. The sensitivity of various ultrasonic parameters in evaluating the performance of asphalt mixtures is also worthy of attention.

Therefore, in this research, four grades of asphalt mixtures were fabricated using a Superpave gyratory compactor (SGC), and the UPP of these asphalt mixtures at the dryness, saturation, and different freeze–thaw cycles were measured. Furthermore, the distribution characteristics of UPP on different graded asphalt mixtures at room temperature were analyzed using the normal distribution. Then, according to the results of the Marshall tests, splitting tests, and compression tests, the correlation between the three ultrasonic parameters and both the volume index of the asphalt mixtures and the mechanical performance indexes was analyzed by using the grey correlation algorithm. The functional relationship between the compressive strength at different temperatures and the UPV was established. Finally, the reliability of predicting high-frequency dynamic modulus by ultrasonic velocity was evaluated. It was expected to be a reference for the evaluation of the performance of asphalt mixtures by ultrasonic parameters. The main aims of the research are as follows:

- To establish the distribution function of the UPP for different asphalt mixtures and evaluate the reliability and sensitivity of three ultrasonic parameters in analyzing the performance of asphalt mixtures.
- To study the variation of three ultrasonic parameters in asphalt mixtures at dry and saturated states at different temperatures so as to provide a basis for formulating a standardized method for the UTT of asphalt mixtures.
- To analyze the correlation between three ultrasonic parameters and the asphalt aggregate ratio, aggregate content, volume indexes, and mechanical performance indexes at different temperatures; to study the internal mechanism affecting the ultrasonic travel speed in asphalt mixtures; and then to establish the functional relationship between the UPV and compressive strength of asphalt mixtures with temperature.
- To deduce the dynamic modulus of four asphalt mixtures at high-frequency load by using UPV and comparing them with the fitting results of the Sigmoid model, and then to analyze the feasibility of using ultrasonic to calculate the high-frequency dynamic modulus of asphalt mixtures.

## 2. Materials and Methods

### 2.1. Materials and Sample Preparation

To study the ultrasonic transmission characteristics of different aggregate gradations of asphalt mixtures in various environments, four grades of asphalt mixtures were prepared by SGC. The gradations of four types of asphalt mixtures, named AC (Suspended dense asphalt mixture), SUP (Superpave graded asphalt mixture), SMA (Skeleton dense asphalt mixture), and OGFC (Skeleton void asphalt mixture), are exhibited in Table 1. Corresponding optimum asphalt aggregate ratios are 4.8%, 4.9%, 5.1%, and 5.5%, respectively. Asphalt mixtures are mainly composed of asphalt, coarse aggregate, fine aggregate, mineral powder, etc. In the fabricating process, high-performance asphalt was adopted as the binder, and its performance parameters are exhibited in Table 2. Basalt aggregate was applied as aggregate to build the skeleton of asphalt mixtures, and limestone mineral powder with a size less than 0.075 mm was adopted as filler.

### 2.2. Testing and Analytical Methods

#### 2.2.1. Strategy of the Research

**Figure 1:**
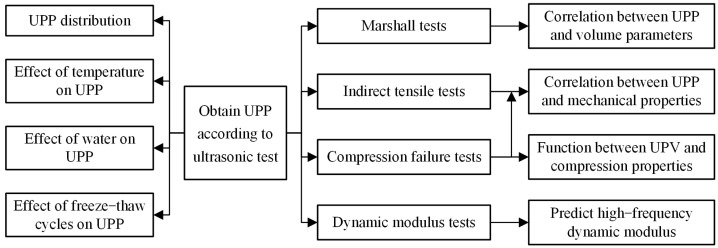
The strategy of this research.

The strategy of this research is shown in Figure 1.

#### 2.2.2. Ultrasonic Tests of Asphalt Mixtures

**Figure 2:**
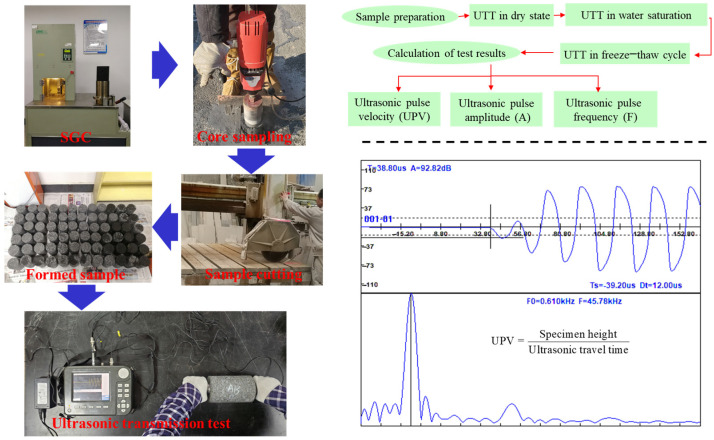
Sample preparation and ultrasonic transmission tests.

To eliminate the influence of the uneven surface of mixture specimens on the UPP, the ultrasonic test specimens are obtained by core drilling sample and cutting. The corresponding sample acquisition and testing processes are shown in Figure 2. The ultrasonic testing instrument is a ZBL-U520/510 non-metallic ultrasonic monitor produced by Beijing Zhibolian Technology Co., Ltd. (Beijing, China). The UPP in four types of asphalt mixtures was measured at the dryness from −20 °C to 60 °C (at 10 °C increments) and at saturated water from 10 °C to 60 °C (at 10 °C increments). In addition, to study the influence of freeze–thaw cycle and damage from UPP, the UPP of four grades of asphalt mixtures with various freeze–thaw cycles was tested at 60 °C water saturation. The freeze–thaw cycle process was −18 °C for 16 h and 60 °C for 8 h.

#### 2.2.3. Splitting and Compression Failure Tests of Asphalt Mixtures

**Figure 3:**
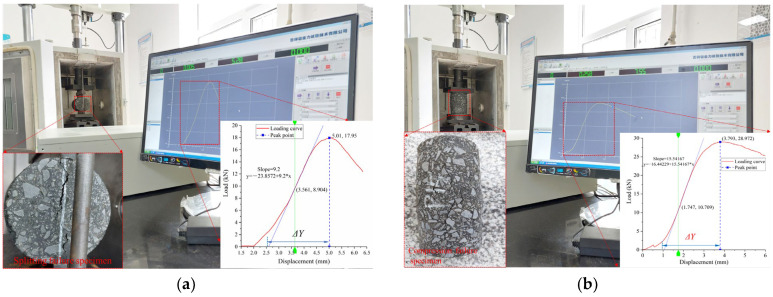
Properties tests of asphalt mixtures: (**a**) splitting test; (**b**) compression failure test.

Splitting and compression tests were implemented to determine the mechanical performance of asphalt mixtures at low, room, and high temperatures. Both tests adopt a displacement loading mode. The loading rate of the splitting test at low temperature (−10 °C) is 1 mm/min, and that of the splitting tests at 20 °C and compression test (from 10 °C to 50 °C at 10 °C increments) is 2 mm/min. The two tests are shown in Figure 3a,b, respectively, and the corresponding mechanical properties are calculated by Equations (1)–(6) [21].

(1) $$\sigma_{I}=0.006287\times P_{I}/h$$

(2) $$\varepsilon_{I}=Y_{I}\times (0.0307+0.0936\times \mu )/(17.94-0.314\times \mu )$$

(3) $$S_{I}=P_{I}\times (3.588-0.0628\times \mu )/(h\times Y_{I})$$

(4) $$R_{C}=4P_{C}/(\pi d^{2})$$

(5) $$\varepsilon_{C}=Y_{C}/h$$

(6) $$S_{C}=R_{C}/\varepsilon_{C}$$

where $\sigma_{I}$ and $R_{C}$ are the indirect tensile strength and compressive strength, respectively; $P_{I}$ and $P_{C}$ are the indirect tensile failure load and compression failure load, respectively; $\varepsilon_{I}$ and $\varepsilon_{C}$ are the indirect tensile failure strain and compressive failure strain, respectively; $Y_{I}$ and $Y_{C}$ are the vertical deformation of indirect tensile failure and compression failure specimens, respectively; h and d are the specimen’s height and diameter, respectively; μ is Poisson’s ratio, 0.25 at −10 °C and 0.35 at 20 °C.

#### 2.2.4. Dynamic Modulus Tests of Asphalt Mixtures

**Figure 4:**
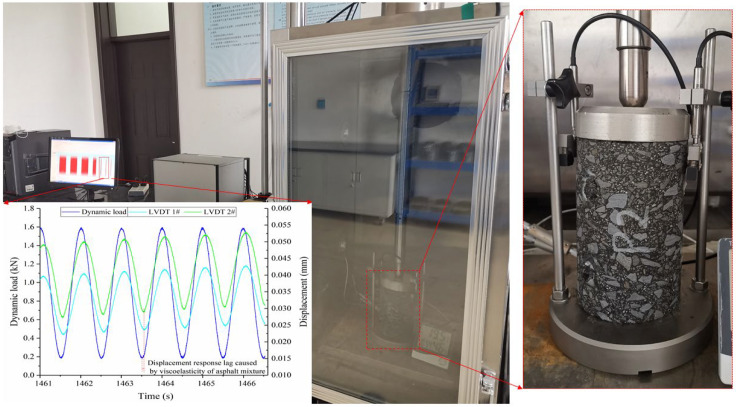
Dynamic modulus testing process of four graded asphalt mixtures.

Relevant research pointed out that the essence of the ultrasonic travel process in asphalt mixtures is the vibration and movement of the high-frequency sound wave, which can reflect the high-frequency dynamic modulus $G^{*}$ of mixtures. The corresponding calculation method is depicted in Equation (7) [22]. According to the viscoelastic theory, $G^{*}$ of mixtures at different frequencies can be constructed into a master curve. To study the applicability of UPV in evaluating the high-frequency $G^{*}$ of asphalt mixtures, the dynamic modulus tests at 10 °C, 20 °C, and 30 °C were carried out [23], and the test’s process is shown in Figure 4, and $G^{*}$ can be deduced by Equation (8).

(7) $$E=v^{2}\rho \frac{\left(1+\mu )(1-2\mu \right)}{\left(1-\mu \right)}$$

(8) $$G^{*}=\frac{4P_{i}h}{\pi d^{2}\Delta Y_{i}}$$

where E is the modulus of mixture deduced by the UPV; ν is the UPV; ρ is the geometric density of mixtures; $P_{i}$ is the mean value of load in the last 5 cycles; $\Delta Y_{i}$ is the mean value of recoverable deformation in the last 5 cycles.

#### 2.2.5. Grey Correlation Analysis of Asphalt Mixtures Properties

The grey correlation analysis method is a method of evaluating the degree of correlation between factors based on the similarity of their development trends, which can effectively compensate for the shortcomings of some linear correlations. The traveling characteristics of ultrasonic pulse in asphalt mixtures are closely related to the volume indexes and mechanical property indexes of asphalt mixtures. Therefore, the grey correlation theory is adopted to evaluate the interrelation between the volume index and the UPP of asphalt mixtures in various environments, and its calculation method is shown in Equations (9) and (10) [24,25].

(9) $$C_{i}=\frac{1}{n}\overset{n}{\underset{k=1}{\sum }}\xi (y_{0}\left(k\right),y_{i}(k))$$

(10) $$\xi \left(y_{0}\left(k\right),y_{i}\left(k\right)\right)=\frac{\underset{i}{\min}\underset{k}{\min}\left|y_{0}\left(k\right)-y_{i}\left(k\right)\right|+0.5\times \underset{i}{\max}\underset{k}{\max}|y_{0}\left(k\right)-y_{i}\left(k\right)|}{\left|y_{0}\left(k\right)-y_{i}\left(k\right)|+0.5\times \underset{i}{\max}\underset{k}{\max}|y_{0}\left(k\right)-y_{i}\left(k\right)\right|}$$

where $C_{i}$ represents the grey correlation grade; $\xi \left(y_{0}\left(k\right),y_{i}\left(k\right)\right)$ is the correlation coefficient between the reference and comparative sequences; $y_{0}\left(k\right)$ is the ultrasonic parameter sequence for reference; $y_{i}\left(k\right)$ are the volume parameters or mechanical property parameters sequence for comparison.

Before the grey correlation analysis, each parameter sequence needs to be normalized. In order not to change the variation characteristics of the original data, the normalization method is given as Equation (11).

(11) $$Y_{i}^{’}=y_{i}(k)/\frac{1}{n}\sum_{k=1}^{n}y_{i}(k)\;k=1,2,\cdots ,n$$

where $Y_{i}^{’}$ is the normalized sequence; $y_{i}(k)$ is the original data, *n* is the length of the sequence.

## 3. Research Results and Discussion

### 3.1. Traveling Characteristics of Ultrasonic Pulse in Mixtures

#### 3.1.1. Distribution of UPP and Influence of Gradation

**Figure 5:**
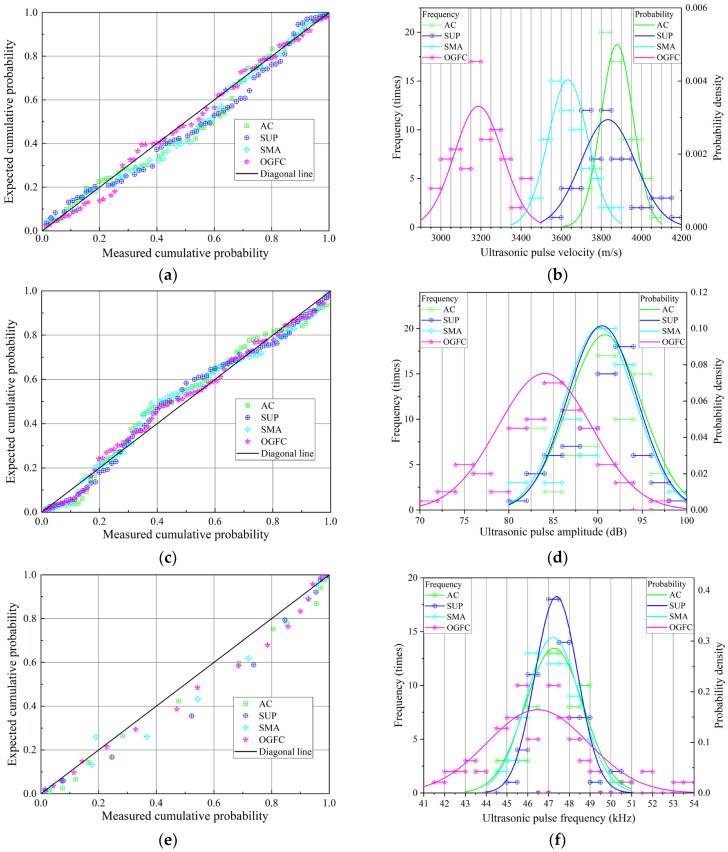
Probability distribution of ultrasonic pulse parameters of asphalt mixtures: (**a**) P–P diagram of pulse velocity; (**b**) frequency and probability distribution of pulse velocity; (**c**) P–P diagram of pulse amplitude (**d**) frequency and probability distribution of pulse amplitude; (**e**) P–P diagram of pulse frequency; (**f**) frequency and probability distribution of pulse frequency.

In the initial study, the UPP, including velocity, amplitude, and frequency of four grades of asphalt mixes at room temperature were tested by the UTT. Because the ultrasonic test is a non-destructive testing technology, a large number of testing data can be collected in the initial test, providing a foundation for the early analysis of the UPP in asphalt mixtures. To analyze the distribution characteristics and discreteness of the three ultrasonic indexes, histograms of the frequency distribution of the three ultrasonic indexes are plotted, as shown in Figure 5. Subsequently, the normal distribution function is adopted to analyze the distribution characteristics of ultrasonic parameters, and the P–P diagram is employed to evaluate the reliability of the normal distribution.

The actual cumulative probability is essentially consistent with the theoretical cumulative probability, as depicted in Figure 5a,c,e, implying that the ultrasonic parameters of the four grades of asphalt mixes tested in the study can better meet the normal distribution. Table 3 describes the normal distribution analysis results of ultrasonic parameters for the four grades of asphalt mixes. As seen in Table 3 and Figure 5b,c,f, there are certain differences in ultrasonic parameters due to differences in gradation type, void ratio (VV), or asphalt content. Furthermore, it was discovered that UPV is sensitive to the gradation change of mixtures, which means that UPV can be better employed to investigate the performance of asphalt mixtures with various aggregate gradations.

Moreover, the UPV of suspension-dense mixtures (AC and SUP) is significantly faster than that of skeleton-dense mixtures (SMA) and skeleton-gap mixtures (OGFC), as shown in Table 3. The amplitude and frequency of the suspension-dense mixture and skeleton-dense mixture are much faster than those of the skeleton-gap mixture. However, when compared to UPV and amplitude, frequency has low sensitivity, implying that frequency is not suitable for characterizing the asphalt mixtures’ performance.

#### 3.1.2. Effect of Temperature on UPP in Four Grades of Mixtures

**Figure 6:**
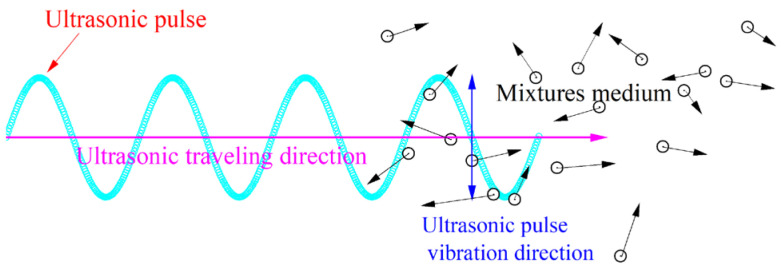
Traveling mechanism of ultrasonic pulse in mixture.

**Figure 7:**
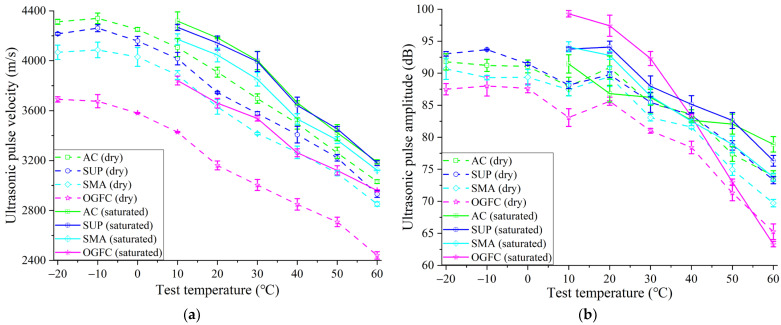
Effects of temperature and dry–wet state on ultrasonic pulse characteristics in asphalt mixtures: (**a**) variation of pulse velocity with temperature; (**b**) variation of pulse amplitude with temperature.

The movement of ultrasonic pulses in asphalt mixtures relies on the particle vibration of the medium, as shown in Figure 6. Testing temperature changes affect an asphalt mixture’s performance, which in turn affects medium particle vibration, which in turn affects the movement of ultrasonic waves in the asphalt mixture. Figure 7 shows the variation of the UPV and amplitude with specimen temperature in four types of asphalt mixtures. The UPV continues to decline with rising temperatures and exhibits a pattern of initially slowing down and then accelerating its decline. According to the findings, asphalt continuously softens as the temperature rises, which prevents sound waves from moving through asphalt mixtures. The performance variety of the asphalt mixture with temperature is reduced at low temperatures, making the UPV less sensitive to variations in the specimen’s temperature.

The attenuation of ultrasonic waves in mixtures can be directly identified by the ultrasonic pulse amplitude. The diffraction and reflection of the ultrasonic pulse are increased in the asphalt mixture due to the voids and viscosity of the asphalt medium, which causes the sound energy of the ultrasonic wave to attenuate during movement. The amplitude is less impacted by the temperature change when the temperature is low. The rapid attenuation of sound energy in the asphalt mixture is brought on by the viscous resistance of the asphalt mixture increasing as a result of a temperature rise. Additionally, it can be seen that the OGFC mixture with the highest void ratio has the greatest ability to dissipate ultrasonic pulse energy and the smallest ultrasonic pulse amplitude for SMA.

Table 4 shows the variation rate of UPV and amplitude with temperature. When the temperature rises from −20 °C to 60 °C at dryness, the UPV of AC, SUP, SMA, and OGFC mixtures decreases by 29.69%, 30.47%, 29.89%, and 33.87%, respectively. The corresponding amplitude decreases by 19.42%, 21.19%, 23.12%, and 25.42%, respectively. It can be found that the UPV and amplitude of OGFC mixtures are most affected by temperature. And it is well known that after the temperature rises, the sound in the air will spread faster. Additionally, the SMA has the highest asphalt content. This means that the influence of temperature on the UPV and amplitude of the asphalt mixture is not only due to the rise of air temperature in the mixture’s voids but also the increase of asphalt viscous resistance. The subsequent researches focuses on the internal factors affecting the transmission of ultrasonic pulse in asphalt mixtures.

#### 3.1.3. Effect of Water on UPP in Four Grades of Mixtures

Because there are some voids in the asphalt mixture, the internal open voids are occupied by water after immersion, which will affect the transmission of ultrasonic pulse in the asphalt mixture. To study the ultrasonic movement characteristics in the asphalt mixture at water saturation, the mixture specimens were first immersed in water to allow them to absorb sufficient water. Then, the UPP of the mixture specimens at water saturation was tested.

Figure 7a shows the UPV of asphalt mixtures at water saturation. The UPV exhibits a decreasing variation at water saturation with an increase in test temperature. In contrast with the UPV results in the dry, it can be found that the UPV of the four types of asphalt mixtures clearly increases after water saturation. The voids in the asphalt mixture are replaced by water, and the UPV in the water is faster than that in the voids, so the ultrasonic transmission for the entire specimen after water saturation is accelerated.

Figure 7b shows the comparison of ultrasonic pulse amplitude in dry and saturated environments. The ultrasonic pulse amplitude after water saturation is higher than in dry conditions. But, this trend fluctuates due to the complicated internal structure of the asphalt mixture, and the variation at various temperatures is noticeably different. Table 4 shows that the UPV of the AC, SUP, SMA, and OGFC mixtures decreases by 26.14%, 25.47%, 25.15%, and 22.95%, respectively, as the temperature increases from 10 °C to 60 °C at water saturation. And the corresponding amplitude decreases by 13.73%, 18.60%, 21.60%, and 36.23%, respectively. As the temperature rises, the decreased value of UPV of asphalt mixture at water saturation is lower than that of dry samples (as shown in Table 4). This might be explained by the fact that void water temperature has a greater impact on UPV than air temperature. When the temperature rises, the disordered movement of the mixture of medium particles intensifies (as shown in Figure 6), which counteracts the orderly directional movement of the ultrasonic waves, resulting in the reduction of the transmitted sound energy, and then the amplitude decreases with the increase in temperature.

The experimental temperature and the dry or wet state of specimens should be strictly controlled when using ultrasonic testing technology to study the performance of asphalt mixtures. Reasonable control variables, particularly in the freeze–thaw cycle testing process, should be considered to obtain reliable test results.

#### 3.1.4. Effect of Freeze–Thaw Cycles on UPP in Four Grades of Mixtures

From the above test results, the test temperature and the dry or wet state of the specimen have a significant impact on the transmission characterization of the ultrasonic pulse. To avoid this influence during the freeze–thaw cycle test, the testing conditions were controlled at 60 °C water saturation.

**Figure 8:**
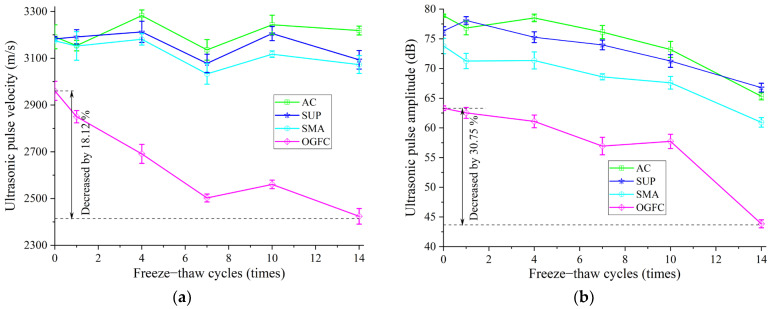
Effect of freeze–thaw cycle on UPP in asphalt mixture: (**a**) variation of pulse velocity with freeze–thaw cycle; (**b**) variation of pulse amplitude with freeze–thaw cycle.

Figure 8 shows the variation of UPV and amplitude for four grades of asphalt mixtures with the number of freeze–thaw cycles. The UPV and amplitude generally show a decreasing trend with an increase in freeze–thaw cycles, and the decreasing range of the OGFC mixture with the largest voids is the most obvious. The UPV and amplitude were both reduced by 18.12% and 30.75% after 14 freeze–thaw cycles, respectively.

Due to the effect of frost-heaving force, the voids in the asphalt mixture increase continuously as the freeze–thaw cycles increase, resulting in water entering the specimen. The transmission speed of ultrasonic waves in water is slower than in aggregate, which can reduce the UPV of the asphalt mixture specimen and significantly increase ultrasonic energy loss, as shown in Figure 8b.

### 3.2. Correlation between UPP and Both Volume Indexes and Performance Indexes

From the above analysis, it can be inferred that the ultrasonic transmission characteristics of mixtures are closely related to the properties of mixtures. To quantitatively analyze the correlation grade, the grey correlation theory is applied to calculate the correlation between the UPP and VV, voids in the mineral aggregate (VMA), voids filled with asphalt (VFA), asphalt content, indirect tensile properties at −10 °C and 20 °C, resistance to compression properties at 20 °C and 50 °C, and aggregate content for each grade. This can lay the foundation for establishing the functional relationship between ultrasonic characteristics and mixture properties.

#### 3.2.1. Correlation between UPP and Volume Parameters

**Figure 9:**
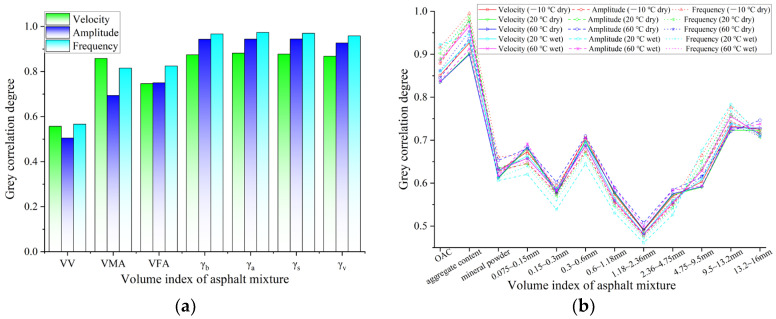
Correlation between UPP and volume parameters of asphalt mixtures: (**a**) correlation between UPP and volume indexes; (**b**) correlation between UPP and aggregate content.

Due to the discreteness of the fabricating process for mixture specimens, there are still some differences in the volume indexes for the same grading of mixture specimens. Therefore, the grey correlation grade between the UPP and the volume indexes (VV, VMA, and VFA), bulk density (*γ_b_*), apparent density (*γ_a_*), apparent dry density (*γ_s_*), and geometric density (*γ_v_*) were calculated, as shown in Figure 9.

The analysis results show that the VMA, VFA, *γ_b_*, *γ_a_*, *γ_s_*, and *γ_v_* of asphalt mixtures have a closer correlation with the UPP than VV. Among them, the correlation degree of the four types of density is the highest, which can also be reflected in Equation (7). There is a functional relationship between the UPV and the density of asphalt mixtures.

Moreover, from the correlation results of VMA and VFA, it can be inferred that there seems to be a close relationship between the UPP and mineral aggregate content and asphalt content. To further analyze and investigate the influence of asphalt and aggregate content on the UPP, the grey correlation between gradation, asphalt aggregate ratio, and the UPP at −10 °C, 20 °C, 60 °C dry environment, and 20 °C and 60 °C water saturation is calculated, as shown in Figure 9b.

Figure 9b shows that the UPP is closely related to the asphalt aggregate ratio and aggregate content of mixtures, which means that the transmission of ultrasonic pulse in asphalt mixtures is mainly affected by the asphalt content and the mineral materials content. Further, the correlation between the aggregate content at each grade and the ultrasonic parameters is analyzed. It can be observed that the UPP has a higher correlation with the mineral aggregate content of 9.5~13.2 mm and 13.2~16 mm. The increase of aggregate content with the large particles in the asphalt mixture will be beneficial to the transmission of the ultrasonic pulse.

#### 3.2.2. Correlation between UPP and Properties Parameters of Asphalt Mixture

**Figure 10:**
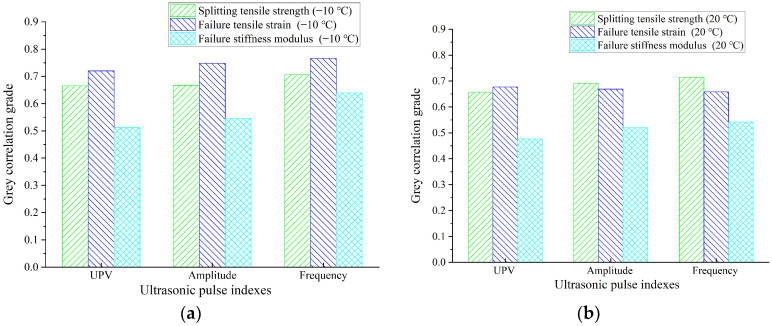

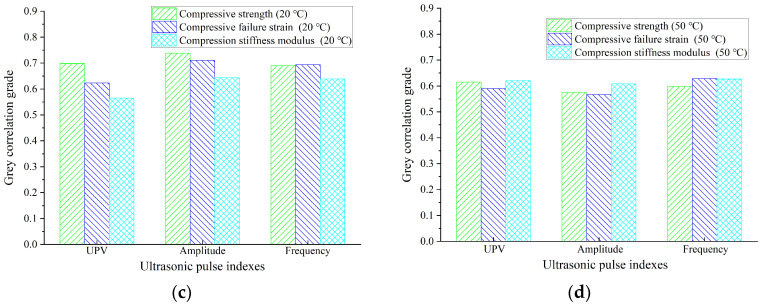
Correlation between UPP and properties parameters of asphalt mixtures: (**a**) correlation with low temperature (−10 °C) performance; (**b**) correlation with splitting performance at room temperature (20 °C); (**c**) correlation with compression performance at room temperature (20 °C); (**d**) correlation with compression performance at high temperature (50 °C).

According to Equation (7), the transmission of an ultrasonic pulse in an asphalt mixture is related to the mechanical properties parameters of the asphalt mixture. To analyze the relationship between the UPP and mechanical properties of asphalt mixtures, the correlation between the UPP and properties parameters of asphalt mixtures at low, normal, and high temperatures is calculated by the grey correlation algorithm. The analysis results are shown in Figure 10.

As can be seen from Figure 10a, the three pulse parameters have a good correlation with splitting parameters at low temperatures (−10 °C), and it can be found that the UPP has a better correlation with low-temperature splitting strength and strain compared with modulus. Similar to the low-temperature properties, the UPP also has a good correlation with the compressive strength parameters and the splitting resistance parameters at room temperature. Moreover, the strength and strain have a higher correlation, as shown in Figure 10b,c. This means that constructing the functional relationship between strength and UPP has good reliability.

It can be seen from Figure 10d that the correlation between UPP and compression parameters at high temperature decreases, which is primarily caused by the asphalt softening at higher temperatures and the rise in factors affecting the transmission of ultrasonic in asphalt mixtures. At room temperature and high temperature, it can be found that the compressive strength has a better correlation with UPP than the compressive strain and modulus. Therefore, the functional relationship between compressive strength and UPV will be established in the subsequent analysis.

#### 3.2.3. Functional Relationship between UPV and Compression Properties

According to the above analysis, the UPP has an excellent correlation with the strength of asphalt mixtures at different temperatures. Moreover, the strength of four kinds of asphalt mixtures changes with the variation of temperature, and the existing relationship (Equation (7)) characterizes the functional relationship between the mixtures’ modulus and the ultrasonic UPV. It is difficult to reflect the relationship between the strength (or modulus) varying with temperature and UPV.

**Figure 11:**
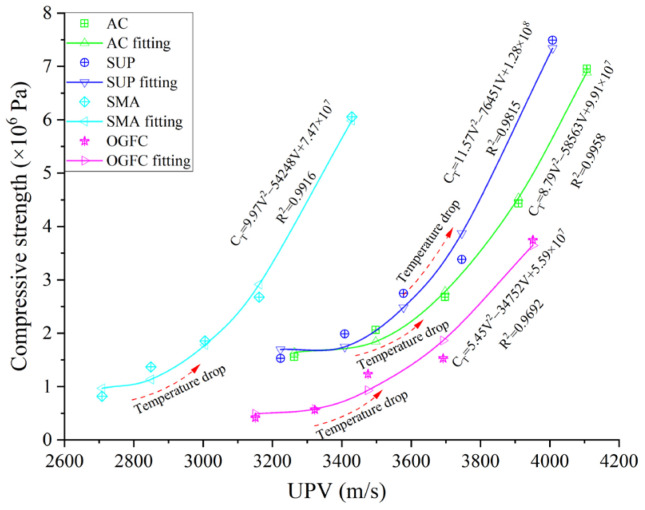
Functional relationship between compressive strength and UPV.

Therefore, in this paper, the compressive strength from 10 °C to 50 °C (at 10 °C increments) was tested by uniaxial compression tests, and a scatter diagram between the UPV and compressive strength at five temperatures was constructed, as shown in Figure 11. Figure 11 shows that with the increase of UPV, the compressive strength of asphalt mixtures shows an approximate parabolic growth from slow to fast. Thus, the quadratic polynomial (Equation (12)) is adopted to build the functional relationship between compressive strength and UPV at dry. The fitting results are shown in Figure 11.

From Figure 11 and the correlation coefficient R^2^, it can be found that the quadratic polynomial can better characterize the quantitative relationship between the UPV (v) and compressive strength (CT) for four grades of asphalt mixtures at various temperatures. However, it can also be clearly found that the fitting parameters of asphalt mixtures with different gradations are quite different. This is mainly because there are great differences in asphalt content and skeleton type for different graded asphalt mixtures, and the influence of temperature change on their compressive strength and UPV is obviously different.

(12) $$E(T)=Av{\left(T\right)}^{2}+Bv(T)+C$$

where *A*, *B*, and *C* are the fitting parameters.

### 3.3. Application of UPV to Predict High-Frequency Dynamic Modulus of Asphalt Mixtures

The transmission of ultrasonic pulse in asphalt mixtures is essentially the vibration transmission of mass points with high-frequency vibration in asphalt mixtures. Relevant studies pointed out that the dynamic modulus at high-frequency loads of asphalt mixtures could be deduced by Equation (7) based on the ultrasonic test results [4]. Therefore, the dynamic modulus tests of four kinds of mixtures at 10 °C, 20 °C, and 30 °C are carried out, and the dynamic master curves of four kinds of mixtures are constructed. The dynamic modulus of four kinds of asphalt mixtures at high-frequency loads is calculated by the Sigmoid model (as shown in Equation (13)) and compared with the dynamic modulus deduced by UPV [26].

(13) $$\log(G^{*})=\left(\log(G_{\max})-\log(G_{\min})\right)/\left[{\left(1+\lambda e^{\beta +\gamma \log\omega }\right)}^{\frac{1}{\lambda }}\right]+\log(G_{\min}),$$

where $\log(G^{*})$ is the logarithm of the dynamic modulus; $\log(G_{\min})$ and $\log(G_{\max})$ are logarithms of static and glassy modulus to be fitted, respectively. λ, β, and γ are the parameters to be fitted to demonstrate the morphological characteristics of the Sigmoid model curve; log(ω) is the logarithm of the angular frequency.

**Figure 12:**
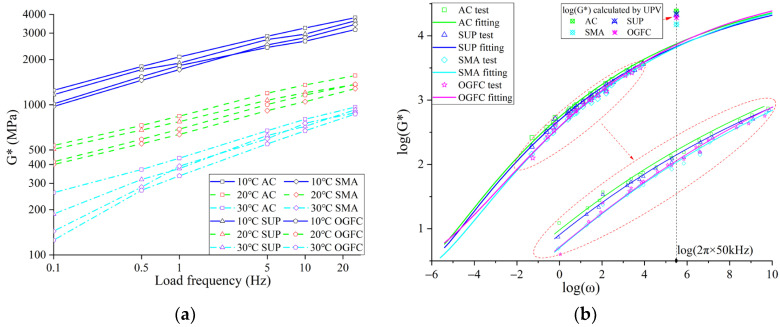
Dynamic modulus test results and main curves fitting comparison: (**a**) dynamic modulus; (**b**) fitting and prediction of dynamic modulus.

Figure 12 shows the change of dynamic modulus of four kinds of mixtures with test temperature and frequency. As can be seen from Figure 11, the dynamic modulus of the mixtures increases with the rise of loading frequency. As the test temperature increases, the dynamic modulus of mixtures decreases.

To further calculate the dynamic modulus of four kinds of mixtures at high-frequency load and compare the dynamic modulus calculated by the Sigmoid model with that calculated by UPV, the main curves of the dynamic modulus of four kinds of mixtures at 20 °C are established. Subsequently, the main curves are fitted by the Sigmoid model (Equation (13)) [27]. The fitting results are shown in Table 5 and Figure 12. Figure 12 illustrates that the Sigmoid model can better characterize the variation of dynamic modulus of asphalt mixtures with loading frequency.

To compare and analyze the relationship between the dynamic modulus calculated by UPV and that obtained by the Sigmoid model, the dynamic modulus of asphalt mixtures at 50 kHz is calculated according to the fitting results, and the error between the dynamic modulus obtained by the two algorithms is calculated, as shown in Table 5. The calculation results in Table 5 indicate that the dynamic modulus at higher frequency can be deduced by using the UPV, but the predictive accuracy of the two algorithms is different for different graded asphalt mixtures, in which the predicting errors of SUP and AC are larger. The dynamic modulus of the OGFC and SMA mixture deduced by using the UPV at a high-frequency load has higher reliability.

## 4. Conclusions

The following conclusions can be distilled from the aforementioned testing and analysis results:

- Ultrasonic traveling parameters in asphalt mixtures present a good normal distribution, in which the UPV is the most sensitive to the gradation change of asphalt mixtures and has obvious advantages in evaluating the performance of asphalt mixtures. The sensitivity of frequency is poor.
- With the increase in temperature, both the UPV and amplitude in dry and wet mixtures samples demonstrate a downward trend. Among them, OGFC mixtures with skeleton-gap structures have the largest decline. The maximum decreases in UPV and amplitude are 33.87% and 36.23%, respectively. The disordered movement of the mixture of medium particles intensifies after the temperature rises, which counteracts the orderly directional movement of the ultrasonic waves, resulting in the reduction of the transmitted sound energy. In addition, compared with other graded mixtures, freeze–thaw cycles also have the greatest impact on OGFC. After 14 freeze–thaw cycles, the UPV and amplitude decreased by 18.12% and 30.75%, respectively.
- Mixture density, asphalt content, and the content of 9.5~13.2 mm and 13.2~16 mm mineral aggregate have a greater impact on ultrasonic parameters. Increasing the content of coarse aggregates can facilitate the transmission of ultrasonic waves in asphalt mixtures.
- The ultrasonic parameters have a good correlation with the mechanical properties of the mixture. According to the quadratic function, the UPV can be applied to predict the compression strength at different temperatures, but the corresponding prediction functions of different mixtures are different. The existing equation can be employed to calculate the high-frequency dynamic modulus by UPV. The dynamic modulus of the OGFC and SMA mixture deduced by using the UPV at a high-frequency load has higher reliability.

## Figures and Tables

**Table 1:** Gradation of four grades of asphalt mixtures.

Gradation Types	Sieve Size									
	0.075 mm	0.15 mm	0.3 mm	0.6 mm	1.18 mm	2.36 mm	4.75 mm	9.5 mm	13.2 mm	16 mm
AC (%)	6	10	13.5	19	26.5	37	53	76.5	95	100
SUP (%)	3	5	12	15	22	35	50	70	91	100
SMA (%)	10	12	13	16	19	20.5	27	62.5	95	100
OGFC (%)	4	5.5	7.5	9.5	12	16	21	70	95	100

**Table 2:** Properties of asphalt binder used by the research.

Properties	Standard Range	Test Value
25 °C Penetration (0.1 mm)	60~80	67.9
Ductility (cm) at 5 °C	≥20	26.8
Softening point TR and B (°C)	≥65	76.4
Elastic recovery (%) at 25 °C	≥85	90.3
After aging test		
Aging mass loss (%)	≤±0.8	0.26
25 °C Penetration ratio (%)	≥60	81.5
Residual ductility (cm) at 5 °C	≥10	14.4

**Table 3:** Normal distribution results of ultrasonic parameters.

Gradation Types	Ultrasonic Pulse Velocity (m/s)		Ultrasonic Pulse Amplitude (dB)		Main Frequency of Ultrasonic Pulse (kHz)	
	*μ*	*σ*	*μ*	*σ*	*μ*	*σ*
AC	3878.33	78.477	90.80	4.121	47.27	1.394
SUP	3833.64	134.737	90.49	3.919	47.38	1.026
SMA	3633.69	97.921	90.23	3.954	47.22	1.295
OGFC	3187.71	119.728	84.04	5.295	46.48	2.425

Note: *μ* and *σ* is the characteristic parameter of the normal distribution.

**Table 4:** The variation rate of UPV and amplitude with temperature.

Gradation Types	Variation Rate of UPV (%)			Variation Rate of Amplitude (%)		
	From −20 °C to 60 °C (Dry)	From 10 °C to 60 °C (Dry)	From 10 °C to 60 °C (Water)	From −20 °C to 60 °C (Dry)	From 10 °C to 60 °C (Dry)	From 10 °C to 60 °C (Water)
AC	29.69	26.16	26.14	19.42	15.89	13.73
SUP	30.47	27.02	25.47	21.19	16.83	18.60
SMA	29.89	26.59	25.15	23.12	20.27	21.60
OGFC	33.87	28.81	22.95	25.42	21.45	36.23

**Table 5:** Fitting results of the Sigmoid model parameters of four asphalt mixtures.

Mixs	Fitting Parameters						Dynamic Modulus Comparison		
	Log (*G_min_*)	Log (*G_max_*)	*β*	*γ*	*λ*	R^2^	Log (Calculated Value of Model)	Log (Calculated Value of UPV)	Prediction Error (%)
AC	0.69	4.938	−0.607	−0.138	−0.846	0.9966	3.877	4.384	11.58
SUP	0.675	4.912	−0.599	−0.136	−0.861	0.9956	3.831	4.336	11.63
SMA	0.45	4.935	−0.548	−0.147	−0.712	0.9967	3.824	4.178	8.47
OGFC	0.433	4.884	−0.471	−0.169	−0.428	0.9942	3.863	4.284	9.84

## Data Availability

Some or all data, models, or codes that support the findings of this study are available from the corresponding author upon reasonable request.

## Abbreviations

UTT	Ultrasonic testing technology
UPP	Ultrasonic pulse parameters
UPV	Ultrasonic pulse velocity
2S2P1D	Two springs, two parabolic elements, and one dashpot
SGC	Superpave gyratory compactor
AC	Suspended dense asphalt mixture
SUP	Superpave graded asphalt mixture
SMA	Skeleton dense asphalt mixture
OGFC	Skeleton void asphalt mixture
VV	Void ratio
VMA	Mineral aggregate
VFA	Voids filled with asphalt
*γ_b_*	Bulk density
*γ_a_*	Apparent density
*γ_s_*	Apparent dry density
*γ_v_*	Geometric density
